# Mitochondrial DNA Damage Does Not Determine *C. elegans* Lifespan

**DOI:** 10.3389/fgene.2019.00311

**Published:** 2019-04-12

**Authors:** Li Fang Ng, Li Theng Ng, Michiel van Breugel, Barry Halliwell, Jan Gruber

**Affiliations:** ^1^Ageing Research Laboratory, Science Division, Yale-NUS College, Singapore, Singapore; ^2^Department of Pharmacology, Yong Loo Lin School of Medicine, National University of Singapore, Singapore, Singapore; ^3^Neurobiology Programme, Life Sciences Institute, National University of Singapore, Singapore, Singapore; ^4^Environmental Science Laboratory, Science Division, Yale-NUS College, Singapore, Singapore; ^5^Department of Biochemistry, Yong Loo Lin School of Medicine, National University of Singapore, Singapore, Singapore

**Keywords:** mitochondrial DNA, DNA damage, lifespan, healthspan, aging, hormesis, quantitative PCR, radiation

## Abstract

The mitochondrial free radical theory of aging (mFRTA) proposes that accumulation of oxidative damage to macromolecules in mitochondria is a causative mechanism for aging. Accumulation of mitochondrial DNA (mtDNA) damage may be of particular interest in this context. While there is evidence for age-dependent accumulation of mtDNA damage, there have been only a limited number of investigations into mtDNA damage as a determinant of longevity. This lack of quantitative data regarding mtDNA damage is predominantly due to a lack of reliable assays to measure mtDNA damage. Here, we report adaptation of a quantitative real-time polymerase chain reaction (qRT-PCR) assay for the detection of sequence-specific mtDNA damage in *C. elegans* and apply this method to investigate the role of mtDNA damage in the aging of nematodes. We compare damage levels in old and young animals and also between wild-type animals and long-lived mutant strains or strains with modifications in ROS detoxification or production rates. We confirm an age-dependent increase in mtDNA damage levels in *C. elegans* but found that there is no simple relationship between mtDNA damage and lifespan. MtDNA damage levels were high in some mutants with long lifespan (and *vice versa*). We next investigated mtDNA damage, lifespan and healthspan effects in nematode subjected to exogenously elevated damage (UV- or γ-radiation induced). We, again, observed a complex relationship between damage and lifespan in such animals. Despite causing a significant elevation in mtDNA damage, γ-radiation did not shorten the lifespan of nematodes at any of the doses tested. When mtDNA damage levels were elevated significantly using UV-radiation, nematodes did suffer from shorter lifespan at the higher end of exposure tested. However, surprisingly, we also found hormetic lifespan and healthspan benefits in nematodes treated with intermediate doses of UV-radiation, despite the fact that mtDNA damage in these animals was also significantly elevated. Our results suggest that within a wide physiological range, the level of mtDNA damage does not control lifespan in *C. elegans*.

## Introduction

Aging affects biological function at molecular, cellular and tissue levels, resulting in progressive deterioration of metabolic processes, reduced resistance to physiological stress and ultimately increased susceptibility to disease and death (Strehler, 1977; Harman, 1981; Cummings, 2007; Vina et al., 2007; Halliwell and Gutteridge, 2015). Deterioration in energy metabolism, in particular, is one of the key features of aging that is evolutionarily highly conserved (Brand, 2000; Roberts and Rosenberg, 2006; Bratic and Trifunovic, 2010; Gruber et al., 2011). Mitochondria are the main site of energy production but they are also an important source of reactive oxygen species (ROS). Mitochondria may therefore be a source as well as a key target of oxidative damage (Lenaz, 1998, 2001; Halliwell and Gutteridge, 2015). The mitochondrial free radical theory of aging (mFRTA) proposes that accumulation of oxidative damage to mitochondria, generated as by-product of normal respiration, contributes causatively to aging (Harman, 1972; Mecocci et al., 1999). Oxidative damage to mitochondrial DNA (mtDNA) is of particular interest in this context, because of its role as carrier of genetic information. Damage to DNA may affect gene expression, impair DNA replication and is an important cause of mutations (Adelman et al., 1988; Loft and Poulsen, 1996; Birch-Machin, 2006; Wang et al., 2006; Cline, 2012; Halliwell and Gutteridge, 2015). Accumulation of mtDNA damage and mutations are therefore potential causes of age-dependent mitochondrial and tissue dysfunction during aging (Shigenaga et al., 1994; Von Zglinicki et al., 2001; Alexeyev et al., 2004). In support of this notion are several studies that have reported a significant accumulation of mtDNA damage with age in human (Mecocci et al., 1993), rat and mice (Hamilton et al., 2001), flies (Agarwal and Sohal, 1994), and *Caenorhabditis elegans* (*C. elegans*) (Gruber et al., 2011). Indirect evidence for a role of DNA damage in aging comes from animal models with disruption in their ROS detoxification systems. For example, mice that lack the cytoplasmic form of the superoxide dismutase enzyme (Cu/ZnSOD) experience increased levels of oxidative damage, as measured in total DNA using HPLC electrochemical detection assay and suffer from short lifespans (Elchuri et al., 2005). Another study shows that mice overexpressing catalase, targeted specifically to mitochondria, had less oxidative damage in total DNA and this was associated with longer lifespan (Schriner et al., 2005). However, damage to mtDNA was not measured in these animals.

Other studies have recently challenged the notion that oxidative damage to mtDNA is causally linked to aging. For example, mice heterozygous for the mitochondrial manganese superoxide dismutase (MnSOD) do not have shortened lifespans, despite the fact that they have been reported to suffer from elevated oxidative DNA damage in both nuclear DNA (nDNA) and mtDNA (Van Remmen et al., 2003). Similarly, *C. elegans* lacking the mitochondrial MnSOD are not short lived (Doonan et al., 2008; Honda et al., 2008; Gruber et al., 2011). However, direct data on mtDNA damage in these animals is sparse. We have previously detected only a statistically insignificant trend toward increased mtDNA damage level in *C. elegans* lacking MnSOD (Gruber et al., 2011). In general, an important caveat to most previous reports is that investigations of pro- or antioxidant interventions and perturbations of ROS homeostasis do not typically report levels of oxidative damage to mtDNA. The lack of data regarding mtDNA damage during aging is predominantly due to methodological issues. The most commonly used methods for the determination of DNA damage, such as high-performance liquid chromatography-electrochemical detection (HPLC-ECD) (Hayakawa et al., 1993; Collins et al., 1996; Van Remmen et al., 2003; Schriner et al., 2005), gas chromatography-mass spectrometry (GC-MS) (Halliwell and Dizdaroglu, 1992), liquid chromatography-mass spectrometry (LC-MS) (Serrano et al., 1996; Gan et al., 2012), immunology methods (Santella, 1999), radiolabeled probes (Reddy, 2000; Laws et al., 2001), or fluorescence-based assays (e.g., versions of the comet assay) (Cadet et al., 1998), cannot be readily used to accurately determine DNA damage levels in mitochondria. This is because only 1% of total cellular DNA is mitochondrial in origin. This means that, to determine mtDNA damage levels accurately, it is necessary to enrich mtDNA approximately 1,000–10,000-fold (e.g., to 99% or at least 90% purity) relative to nDNA (Lim et al., 2005). Purification procedures at this level require large amounts of starting material, is labor and time intensive and, most importantly, can lead to artifacts because mtDNA may be oxidized during purification (Senturker and Dizdaroglu, 1999; Lim et al., 2006). These issues limit the applicability of most common DNA damage methods and may explain why, despite its importance, mtDNA damage burden is so rarely reported. A way to overcome these challenges is to employ sequence-specific PCR-based approaches that do not require as much sample purification. Meyer et al. (2007) have reported an elegant method based on qRT-PCR that can be applied to mtDNA. Fundamentally, this DNA damage method is based on the fact that many forms of DNA damage (including strand breaks, cross-links and bulky DNA adducts) can interfere with DNA polymerase progression, thus preventing PCR amplification and resulting in an apparent decrease in the amount of PCR template (Govan et al., 1990; Meyer et al., 2007; Edwards, 2009). This effect can be used to quantify DNA damage by comparing relative amplification of a short DNA fragment (S-fragment) versus that of an extra-long DNA fragment (XL-fragment), designed to be located within the same target sequence. Because the probability of encountering a blocking DNA lesion increases exponentially with the length of the template DNA being amplified (Meyer, 2010), the amplification of the XL-fragment is exponentially more sensitive to DNA damage than that of the S-fragment. During qRT-PCR amplification, this manifests as an apparent difference in the DNA copy number of the XL versus the S amplicon (Santos et al., 2002; Meyer et al., 2007). Because the S sequence is contained within the XL sequence, the actual copy number for the XL and S template is identical and the true ratio between S and XL template is therefore equal to one. However, in the presence of significant DNA damage, the apparent copy number of the XL fragment will decrease, due to DNA damage rendering some of the original XL template non-amplifiable. The observed ratio between the apparent copy number of the XL versus the S fragment can then be converted to DNA lesion frequency using the Poisson Equation (Santos et al., 2002; Meyer et al., 2007; Hunter et al., 2010). We have previously adapted this assay for quantification of specific forms of oxidative mtDNA damage by employing a pre-digest step of DNA with specific DNA repair enzymes (Gruber et al., 2011). Here we report an investigation of the role of mtDNA damage in aging of nematodes using a modified version of this damage assay. The main difference between the two versions of the assay is that we have omitted any pre-incubation of DNA samples with DNA processing enzymes. Some types of DNA lesions such as 8-hydroxy-2′-deoxyguanosine (8-OHdG) are unlikely to interfere with PCR amplification and omitting the pre-digest step therefore reduces sensitivity toward these types of DNA damage. However, we have found that pre-incubation of mtDNA extracts for extended periods of time can induce artifacts and may result in DNA degradation, both of which increase variability. We have therefore removed the pre-digest step in this study, although this mean that it is no longer specific for oxidative damage.

We first explored the sensitivity of our new, sequence specific S-XL-qRT-PCR DNA damage assay by exposing the *C. elegans* to exogenous DNA damage using either UV- or γ-radiation. DNA lesions resulting from UV-irradiation include cyclobutane pyrimidine dimers (CPDs), 6-4 photoproducts (6-4PPs), 8-oxoguanine (8-OxoG), strand breaks, DNA crosslink and DNA-protein crosslinks (Mitchell and Nairn, 1989; Britt, 1995; An et al., 2011; Studer et al., 2012). Many of these DNA lesions, in particular bulky adducts, strand breaks and cross-links inhibit PCR amplification (Govan et al., 1990; Kalinowski et al., 1992), making them easily detectable by q-PCR based assays.

Mitochondria lack the nucleotide excision enzymes needed to repair UV-induced DNA damage (Alexeyev et al., 2013), and are therefore unable to remove or repair most forms of UV-induced DNA damage (Balajee and Bohr, 2000; Hanawalt, 2002; Meyer et al., 2007; Leung et al., 2013). Indeed, we have recently found no evidence for any repair or removal of UV-induced DNA damage in the mtDNA of *C. elegans* (Lakshmanan et al., 2018). Because of the persistence of UV-lesions in mtDNA and because of the type of DNA lesions generated, we expected UV-induced damage to be comparatively easy to detect using our PCR-based method (Meyer et al., 2007).

In contrast to UV-radiation, γ-radiation is more energetic, penetrates tissues more easily and also differs in the predominant types of DNA damage it causes. Exposure to γ-radiation creates many different types of DNA damage, including single-and double-strand breaks, abasic sites, oxidative modifications and DNA-protein crosslinks (Henner et al., 1982; Min et al., 2003; Sudprasert et al., 2006). γ-radiation is a potent inducer of mutations (Borek, 2004) with potential impact on aging dynamic of mtDNA mutations. These types of DNA damage and the resulting mutations may be more similar to endogenous, ROS-mediated DNA damage and age-associated mutations than UV-induced damage. However, DNA lesions generated by either γ- or UV- irradiation can both cause genetic instability and cell death (Min et al., 2003). We therefore tested our assay against endogenous damage generated by both forms of challenge.

We used our method to determine levels of mtDNA damage in wild type *C. elegans* during normal aging. We further explore the role of mtDNA damage in a long-lived mutant strain and in strains with modifications in ROS detoxification or production rates. In addition to these correlative data, we also introduced and quantified exogenous DNA damage to the mtDNA of young animals and followed these animals throughout life to determine the lifespan and healthspan consequences of such perturbations.

## Materials and Methods

### General Maintenance of *C. elegans*

The *C. elegans* strains: the Bristol N2 (wild type), JK1107 (*glp-1*), GA480 (*sod-2/-3*), OK2040 (*mpst-1*), TK22 (*mev-1*), and CB1370 (*daf-2*) were used in this study and were obtained from the Caenorhabditis Genetics Centre (University of Minnesota, Minneapolis, MN, United States). All *C. elegans* strains were grown on nematode growth medium (NGM) agar plates at 20°C except for JK1107 (*glp-1*) strain, which was grown at 25.5°C to prevent progeny. Preparation of NGM agar plates was as previously described in Stiernagle (2006). All experiments used day 4 post-hatching age-synchronized nematode obtained by hypochlorite bleaching.

### Blinded Lifespan Studies

Lifespan of *C. elegans* was observed as described previously under blinded conditions to eliminate experimental bias (Schaffer et al., 2011). The numbers of alive and dead worms were scored. The surviving worms were transferred to fresh plates every 2 days until the post egg-laying period. Worms that failed to move in response to mechanical prodding were scored as dead. Worms that died due to crawling off the plates were censored.

### Blinded Healthspan Studies

Locomotion activity of the worms were scored following the previously reported scoring framework (Herndon et al., 2002). The locomotion activity of the worms was classified into three classes: Class A, B, and C. Class A animals were healthy and moved spontaneously, class B animals moved in respond to prodding, produced non-sinusoidal tracts while class C animals moved their head and/or tail only.

### Relative Distance Traveled

The relative distance traveled of 10–20 worms of each treatment condition was measured and analyzed under blinded conditions. A mark was made just at the border of the bacterial lawn to indicate the starting point of distance to be traveled. In general, individual worms were transferred onto the marking on the plate and allowed to travel for 15 min. Worms were removed and a single photograph of the whole NGM agar plates was captured using a calibrated Leica MZ10F microscope (Leica, Singapore). The line tool provided by the Leica Application Suite Software (v2.6.0 R1) was utilized to measure the distance traveled by the worms.

### Mitochondrial DNA Extraction

MtDNA was extracted as described elsewhere (Yasuda et al., 2006) with several modifications. About 10,000 worms were homogenized in isolation buffer. Debris and nuclei were removed via differential centrifugation at 600 g for 10 min at 4°C. The pellet was discarded and the supernatant was centrifuged at 7200 g for 10 min at 4°C to obtain mitochondria pellet. DNA from crude mitochondria was purified using Prepman Ultra Sample Preparation Reagent (Applied Biosystems) according to manufacturer’s protocol.

### Sequence-Specific Mitochondrial DNA Damage Quantification

MtDNA copy number of a reference sample was quantified using mtDNA copy number method as described in Gruber et al. (2011) and Poovathingal et al. (2012). Briefly, mtDNA copy number of individual nematodes was determined by quantitative real-time PCR (qRT-PCR) amplifying a 71 bp region of the *C. elegans* mitochondrial genome using the worm lysate as the source of DNA template and the mtDNA (short fragment) primers and probe [Forward primer: 5′-GAG CGT CAT TTA TTG GGA AGA AGA-3′ (nucleotides 1838–1861 mtDNA); Reverse primer: 5′-TGT GCT AAT CCC ATA AAT GTA ACC TT-3′ (nucleotides 1883–1908 mtDNA) and Probe: 5′-FAM-AAA ATC GTC TAG GGC CCA C-3′ (nucleotides 1863–1881 mtDNA)]. The probe was labeled with a specific reporter (FAM-labeled) and has non-fluorescent quencher (MGB Probes). This assay was performed using a reference sample of known copy number (quantified by serial dilution generating actual rather than relative copy numbers).

After determining the mtDNA copy number of the reference sample, Sequence-specific mtDNA damage quantification was performed as described elsewhere (Meyer et al., 2007; Gruber et al., 2011) using GeneAMP XL PCR kit (applied Biosystems). Sequence-specific mtDNA damage was quantified in a 6.3 kbp region of the mitochondria genome using sybr green dye with previously reported primers sequence (Hulbert et al., 2007) (Forward primer: 5′-TCG CTT TTA TTA CTC TAT ATG AGC G-3′ (nucleotides 1818–1842 mtDNA), Reverse primer: (5′-TCA GTT ACC AAA ACC ACC GAT T-3′ (nucleotides 8111–8090 mtDNA) and 71 bp region using Taqman probe with forward primer and reverse primer. The amplification factor was determined for each primer sets used for mtDNA damage assay (Gruber et al., 2011). Under ideal conditions, each DNA molecule would double in each PCR cycle. However, this is not necessarily the case, especially for long extension PCR. Different samples, experimental set-ups, reagents and fluorescence dyes can affect PCR performance, resulted in different amplification factors.

The aliquots from the same DNA sample was amplified using XL primers and SYBR Green I dye or using S primers and Taqman probe. DNA lesion frequency was then calculated using the Poisson equation as described in Govan et al. (1990), Britt (1995), and Hunter et al. (2010).

### Comet Assay

*C. elegans* embryonic cells were prepared from eggs isolated from young gravid adults as described in Bianchi and Driscoll (2006), Strange et al. (2007), and Sobkowiak and Lesicki (2009). Briefly, large quantities of gravid adult worms were lysed using egg isolation solution. The lysis reactions were stopped by adding egg buffer solution. The egg pellet was separated from worms’ debris by flotation on a sucrose solution, eggs were then washed to remove sucrose. 1 U/ml Chitinase was added to egg buffer to digest egg shells and to obtain single cell suspension. The dissociated cell suspension was then plated onto Lab-Tek II-CC2 chamber slide (Nunc, Thermo Fisher Scientific Inc.) and kept in a humidified incubator for cell differentiation. After 24 h, the degree of DNA damage was determined using the alkaline comet assay according to Olive and Banath (2006) and Sobkowiak and Lesicki (2009). Briefly, 1% low melting point agarose was added on top of the first layer of agarose contained cells and was allowed to solidify. The microscope slide was then immersed in ice-cold alkaline lysing solution for at least 1 h at 4°C in the dark. After lysis, the microscope slide was placed in a horizontal gel electrophoresis chamber filled with electrophoretic buffer. Electrophoresis was conducted at 25 V and 300 mA for 10 min in a chamber cooled on ice. The slide was then rinse with neutralization solution, dried at room temperature and stained with SYBR Green I. The microscope slide was examined using confocal fluorescence microscope (LSM 510 Carl Zeiss, Jena, Germany) and comets were analyzed using image analysis software, CometScore (TriTek Corp, United States).

### Statistical Analysis

GraphPad Prism version 5.02 for Microsoft Windows (GraphPad Software, San Diego, CA, United States) was used for statistical analysis. Lifespan and healthspan curves were analyzed by plotting Kaplan–Meier survival curves and by conducting Log-rank tests. Mean lifespan data was compared using Log-rank test with appropriate correction for multiple comparisons OASIS 2. All other data were plotted as means ± SEM, analyzed using ANOVA and Bonferroni’s multiple comparisons post-test unless otherwise stated. Differences with *P* < 0.05 were considered as statistically significant. In the figures, *p* values > 0.05 are summarized as ns, *p* values ≤ 0.05 are summarized as one asterisk, *p* values ≤ 0.01 are summarized as two asterisks, *p* values ≤ 0.001 are summarized as three asterisks, and *p* values ≤ 0.0001 are summarized as four asterisks.

## Results

### Validation of the Real-Time PCR-Based Assay for Sequence-Specific mtDNA Damage

**Diagram 1:**
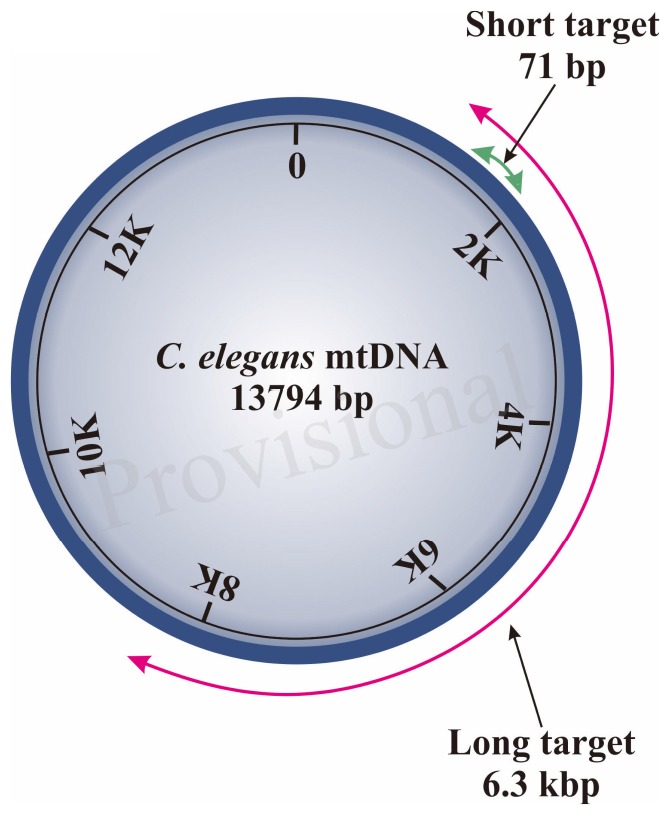
Schematic diagram of region amplified by short and long target using the S-XL-qRT-PCR DNA damage assay within *C. elegans* mitochondrial genome. The short target (71 bp) is represented by a green arrow and the long amplicon (6300 bp) is represented by the red arrow. The short target is too short and is much less affected by DNA damage, therefore can be used as an approximation for the undamaged DNA while the long target is used for DNA quantification as the probability of encountering DNA lesions increases with template size and this long amplicon amplifies the majority of the *C. elegans* mitochondrial genome.

Our S-XL-qRT-PCR assay is based on amplification of a short (S:71 bp) fragment relative to an extra-long (XL: 6.3 kbp) fragment, both within the same mtDNA sequence (Diagram 1). This extra-long target sequence was selected because this 6.3 kbp of mtDNA fragment covers almost half of the 13.7 kbp *C. elegans* mitochondrial genome. Under normal physiological conditions, the short fragment is too short for its amplification to be significantly affected by DNA damage and it thus serves as a reference for internal copy number normalization. Assuming that DNA lesions are randomly distributed within the mtDNA molecule, lesions frequency in a DNA sample can be calculated based on real-time amplification of the extra-long fragment relative to the short fragment (Meyer et al., 2007; Hunter et al., 2010) in the *C. elegans* mitochondrial genome using the Poisson equation (Govan et al., 1990; Britt, 1995; Hunter et al., 2010).

### S-XL-qRT-PCR DNA Damage Assay Detects Dose-Dependence Increase in mtDNA Lesion Frequency in Nematodes Exposed to UV-Radiation

**Figure 1:**
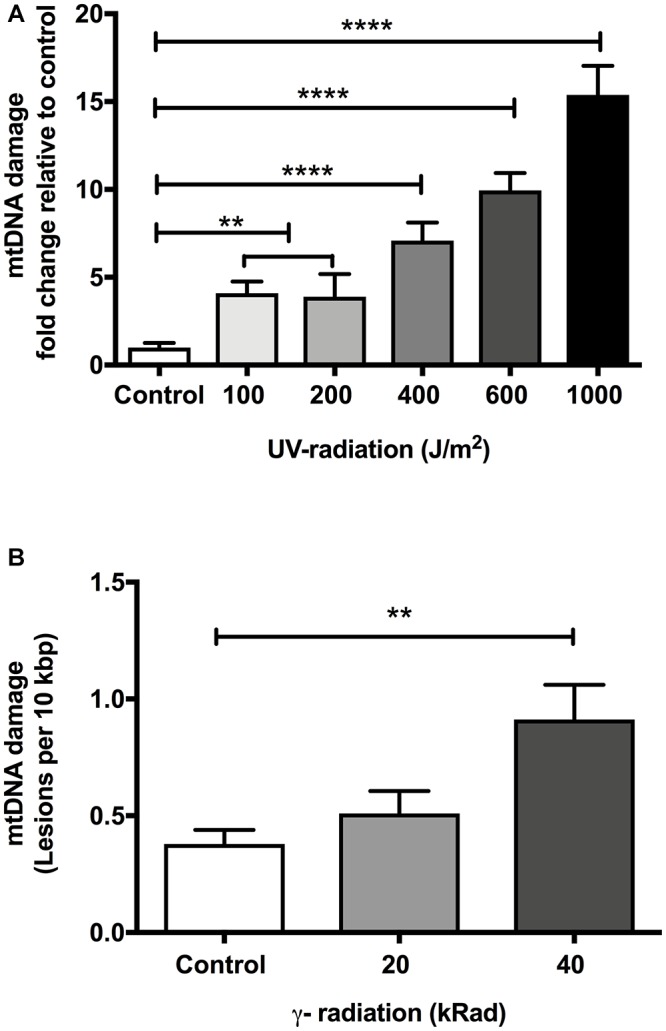
The effect of dose-dependent UV- and γ-irradiation on induction of DNA damage in mitochondria of Day 4 young *glp-1 C. elegans*. **(A)** UV-radiation dose values given to nematodes ranging from 0 to 1000 J/m^2^. Overall, there was a significant dose-dependent increase in the damaged mtDNA in animals exposed to UV-radiation (*p* < 0.0001, One-way ANOVA, *n* = minimum 3 independent experiments). 100 and 200 J/m^2^ had no significant effect on the level of damaged mtDNA relative to untreated control animals (*p* > 0.05, One-way ANOVA with Bonferroni’s post-test). Exposure of nematodes to 400–1000 J/m^2^ UV-radiation significantly elevated the DNA lesions in the nematodes (*P* < 0.0001 for all other conditions, One-way ANOVA with Bonferroni’s post-test). **(B)** Nematodes were exposed to increasing doses of γ-radiation ranging from 0 to 40 kRad. There was a statistically significant elevation in the extent of mtDNA damage (*p* < 0.0001, One-way ANOVA, *n* = minimum 3 independent experiments). The level of damaged mtDNA in nematodes exposed to 20 kRad γ-radiation was at 0.5 lesions per 10 kbp from a baseline level of 0.4 lesions per 10 kbp in non-irradiated control animals, the change in the mtDNA damage level was not statistically significant (*p* > 0.05, One-way ANOVA with Bonferroni’s post-test). Relative to control nematodes, 40 kRad γ-radiation significantly elevated the mtDNA damage levels as evaluated by our S-XL-qRT-PCR DNA damage assay (*p* < 0.01, One-way ANOVA with Bonferroni’s post-test).

Because UV-mediated DNA damage is persistent and of a type that should be reliably detectable using our assay, we used UV-irradiation for initial test of reliability and sensitivity of our assay. In human, UVC exposure below 200 J/m^2^ typically does not cause significant erythemal response (sunburn) (Hemminki et al., 2001; D’Orazio et al., 2013; Diffey and Farr, 2017). In *C. elegans*, Meyer et al. (2007) have shown that DNA damage in mtDNA can be detected with exposure as low as 100 J/m^2^ UV-radiation. Our own preliminary experiments showed no immediate detrimental effects at this level in *C. elegans* (data not shown). We therefore chose 100 J/m^2^ as the lower end of UVC challenge studies. Meyer et al. (2007) found that nematodes exposed to 400 J/m^2^ UVC resulted in up to 10-fold elevation in mtDNA damage, suggesting that 400 J/m^2^ causes significant damage. To compare the sensitivity and reproducibility of our assay, we exposed *glp-1 C. elegans* to different doses of UVC-radiation (254 nm wavelength) ranging from 100 to 1000 J/m^2^. We analyzed samples at 100, 200, 400, 600, and 1000 J/m^2^ UV-radiation to determine the levels of damaged mtDNA relative to controls using our S-XL-qRT-PCR DNA damage assay. As expected, mtDNA damage increased significantly as a function of increasing UV-irradiation dose (Figure 1A, *p* < 0.0001, One-way ANOVA). We were unable to show a statistically significant difference in the mtDNA damage at the lowest levels in nematodes exposed to 100 and 200 J/m^2^ UV-radiation individually (Figure 1A, *p* > 0.05, One-way ANOVA with Bonferroni’s post-test). However, pooling data from the two lowest levels of UV-radiation (100 and 200 J/m^2^), we found significantly elevated mtDNA damage level compared to the non-irradiated control animals (Figure 1A, *p* < 0.01, One-way ANOVA with Bonferroni’s post-test). Levels above 200 J/m^2^ resulted in robust elevation of mtDNA damage level, with 7-, 10-, and 15-fold increases in the mtDNA damage relative to untreated control animals in nematodes exposed to 400, 600, and 1000 J/m^2^ UV-radiation, respectively (Figure 1A, *p* < 0.0001, One-way ANOVA with Bonferroni’s post-test). These UV sensitivity experiment demonstrates that our S-XL-qRT-PCR DNA damage assay is able to sensitively and reproducibly detect UV-induced lesions over a relatively wide range, with significant elevation even at low dose (100 and 200 J/m^2^) of UV-radiation.

### Validation of S-XL-qRT-PCR mtDNA Damage Assay in γ-Irradiated Animals Using Nuclear DNA (nDNA) Lesions Comet Assay

We next tested our assay against DNA damage induced by γ-radiation. Exposure to γ-radiation damages DNA both directly (generating single-and double-strand breaks) (Henner et al., 1982; Min et al., 2003; Sudprasert et al., 2006) and indirectly by the generation of free radicals and ROS, e.g., through radio-lysis of water (Dizdaroglu, 1992; Borek, 2004; Halliwell and Gutteridge, 2015).

In *C. elegans*, γ-radiation doses of less than 1 kRad have previously been reported to have no immediate effect on survival or reproduction rate (Buisset-Goussen et al., 2014), indicating that γ-radiation at this dose is insufficient to kill even the rapidly dividing cells of the germline. The *C. elegans* soma consists mainly of non-dividing cells, known to be more resistant to DNA damage than dividing cells. This means *C. elegans* can typically tolerate higher levels of damaged DNA and is more resistance to radiation than most animals, including humans, that are dependent for their survival on active cell division (Flemming et al., 2000; Bailly and Gartner, 2011). Consistent with this expectation, Johnson and Hartman (1988) have previously reported that radiation doses of more than 100 kRad are required to observe significant lifespan reducing effect in young adult *C. elegans*.

**Figure 2:**
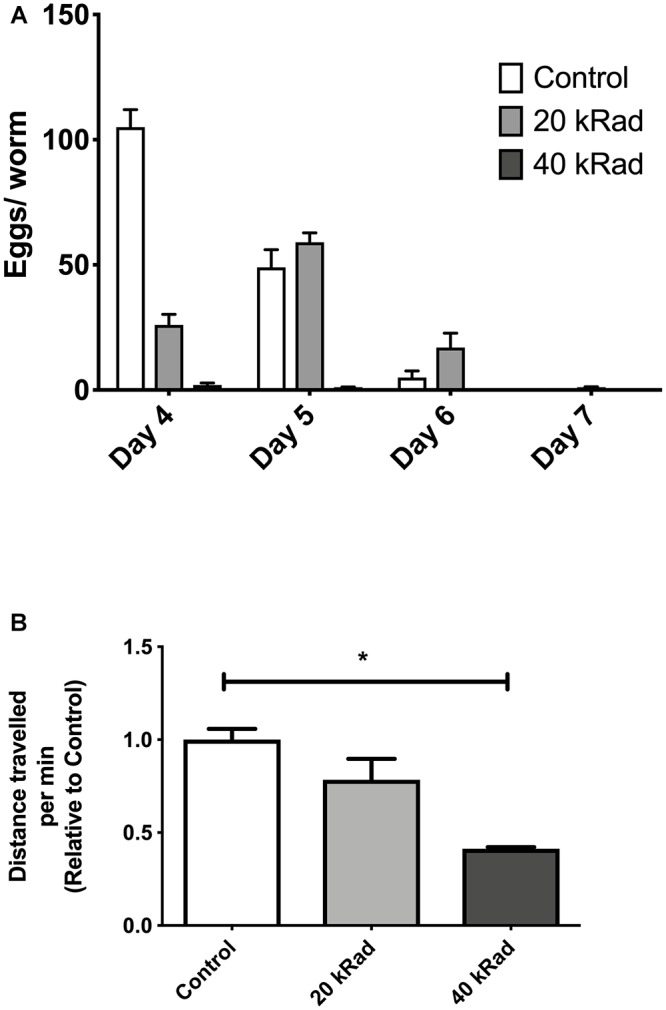
Pilot experiments of various doses of γ-radiation. **(A)** Egg-laying study comparing number of eggs laid by *C. elegans* exposed to 0 (control), 20 and 40 kRad γ-radiation doses. Animals exposed to γ-radiation laid less eggs than non-irradiated control animals. **(B)** Distance traveled by *C. elegans* exposed to 0 (control), 20 and 40 kRad γ-radiation doses. Non-irradiated animals were healthy and active. Animals exposed 40 kRad of γ-radiation explored less than 20 kRad and control.

We initially performed pilot experiments to characterize phenotypes other than survival of nematodes exposed to γ-radiation. We observed that control nematodes that never experienced γ-irradiation appeared healthier, had more offspring and explored more of their plates than γ-irradiated nematodes. Animals exposed to 40 kRad of γ-radiation explored significantly less and has substantially reduced fecundity while those exposed to 20 kRad were less severely affected (Figure 2A,B). We therefore exposed young adult nematodes to either 20 or 40 kRad of γ-radiation, significantly below the level that causes rapid death but well above the level sufficient to kill germline cells and resulting in detectable detriments and therefore expected to induce significant DNA damage to both nDNA and mtDNA.

As shown in Figure 1B, our S-XL-qRT-PCR DNA damage assay was able to detect the elevation of mtDNA damage level in animals challenged with γ-radiation. There was a dose-dependent increase in DNA damage with increasing amount of γ-radiation (*p* < 0.001, One-way Anova). Post-test analysis revealed that while we were able to detect a trend toward higher mtDNA damage in the nematodes challenged with 20 kRad of γ-radiation, this increase in the mtDNA damage was not statistically significant (Figure 1B). However, nematodes exposed to 40 kRad of γ-radiation showed significant elevation of mtDNA damage levels by approximately twofold compared to untreated control animals (*p* < 0.01, One-way ANOVA with Bonferroni’s post-test). These data indicate that the S-XL-qRT-PCR DNA damage assay is also able to detect DNA lesions induced by γ-radiation, although with lower sensitivity compared to UV-radiation.

To further evaluate the sensitivity of the S-XL-qRT-PCR DNA damage assay, we next compared its ability to detect DNA lesions caused by γ-radiation to a well-established assay that is commonly used in γ-irradiated animals, the comet assay.

The comet assay is a single cell electrophoresis method, used in radiation biology to quantify nDNA damage (Dusinska and Collins, 2008; Meyer, 2010). Briefly, cells are embedded in agar and exposed to an electrical field. DNA is drawn toward the anode, forming a comet-like image when viewed under a fluorescence microscope (Gedik et al., 1992). DNA containing single or double strand DNA breaks has a higher mobility in agar and during electrophoresis move faster and further (Collins et al., 2008). The amount of DNA within the comet tail therefore correlates to the extent of DNA damage (Collins et al., 2008). While the comet assay is designed to detect γ-induced DNA strand breaks (Collins et al., 1996) in nDNA of single cells, our PCR assay is designed to detect mtDNA damage. However, γ-radiation will penetrate cells and organelles, inducing DNA damage to both nDNA and mtDNA by direct interaction with DNA and through radiolysis of water in the path of radiation. Increased dose-dependent DNA damage is therefore expected to result in both the mitochondrial and nuclear compartments. We exposed young adult nematodes to either 20 or 40 kRad of γ-radiation and compared the resulting γ-induced DNA damage in nDNA and mtDNA as measured by the comet assay and our S-XL-qRT-PCR, respectively.

**Figure 3:**
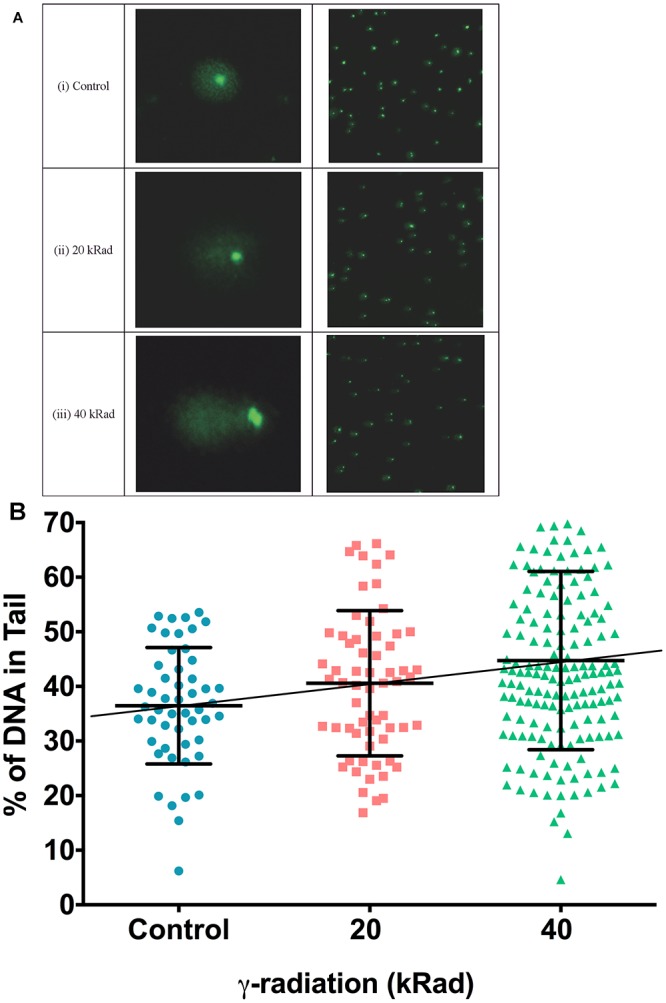
DNA strand breaks induced in γ-irradiated wild type N2 *C. elegans* measured using the comet assay. **(A)** Typical comet images of wild type N2 *C. elegans* embryonic cells obtained from **(i)** untreated control animals (undamaged DNA sample). **(ii)** *C. elegans* exposed to 20 kRad γ-radiation (damaged DNA sample) and **(iii)** *C. elegans* exposed to 40 kRad γ-radiation (damaged DNA sample). **(i)** In undamaged DNA samples, the DNA remains intact within the highly organized structure and is confined to the nucleus, resulting a halo-like structure. **(ii,iii)** When DNA is damaged, the relaxed DNA expands out from the nucleoid during electrophoresis, resulting in a structure that resembles a comet with a head composed of intact undamaged DNA and a tail that consists of damaged/broken fragments of DNA. **(B)** Effects of γ-radiation on DNA damage on nematodes exposed to 0, 20, 40 kRad γ-radiation, determined using the comet assay as the percentage of DNA in the tail. There were 36, 41, and 45% of DNA in tail of the comets in the control, 20 and 40 kRad γ-irradiated animals, respectively. There was a linear relationship between radiation dosage and DNA in tail (R-squared = 1.00). *n* = minimum 55 comets analyzed per condition.

Figure 3Ai–iii are typical comet images of wild type N2 *C. elegans* embryonic cells obtained from untreated control compared to cells from nematodes irradiated with 20 or 40 kRad γ-radiation, respectively. As expected, cells from γ-irradiated nematodes on average have higher comet tail intensity (more damaged DNA) compared to less-damaged untreated controls. The amount of migrating DNA in the comet tail increases in a dose-dependence manner following exposure to γ-radiation (Figure 3B, *p* < 0.01, One-way ANOVA). However, similar to our PCR-based mtDNA damage assay, post-test reveals that the percentage of DNA in the comet tail of the nematodes challenged with 20 kRad γ-irradiation showed a trend to increased damage but this increase individually is not significant relative to the non-irradiated control nematodes (Figure 3B, *p* = 0.08, Student’s *t*-test). However, the percentage of increased of damaged DNA in the comet tail following 40 kRad γ-irradiation was 45% higher, and this change was statistically significant relative to non-irradiated control nematodes (Figure 3B, *p* < 0.001, One-way ANOVA with Bonferroni post-test).

**Figure 4:**
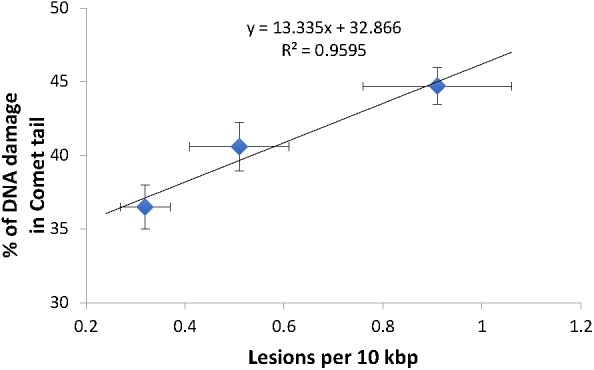
High correlation between the DNA damage level from both comet assay and S-XL-qRT-PCR assay. Both comet assay and S-XL-qRT-PCR DNA damage assay are able to detect significant dose-dependent increase in DNA damage in animals irradiated with 0, 20, and 40 kRad γ-radiation. Each point is a mean of minimum 55 comets (for data plotted in Y-axis) or minimum of 12 replicates (for data plotted on X-axis).

Comparison of mtDNA damage frequency as detected using our S-XL-qRT-PCR DNA damage assay in mtDNA (Figure 1B) to the increase in nDNA strand breaks as detected using the comet assay (Figure 3B), shows a high degree of correlation between the increases in DNA damage in nDNA and mtDNA damage (Best fit R-squared = 0.96) (Figure 4). These data show that both assays are able to detect the increased in damaged DNA at the same level of γ-irradiation. Our S-XL-qRT-PCR DNA damage assay is therefore sensitive toward both UV- and γ-radiation induced mtDNA damage at physiologically relevant levels and its sensitivity for γ-radiation is comparable to that of the comet assay. Next, we therefore applied this tool to explore the role of mtDNA damage in the aging process of *C. ele*gans.

### Does mtDNA Damage Contribute to Aging?

**Figure 5:**
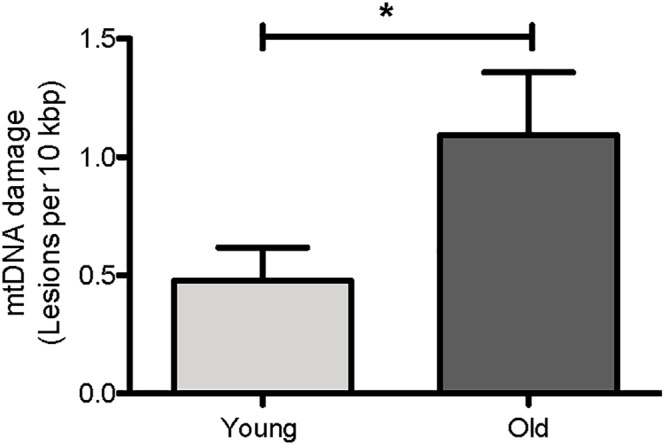
Age-dependent changes in the mtDNA damage level in nematodes. Using our S-XL-qRT-PCR DNA damage assay, there were a statistically significantly more mtDNA lesions in old nematodes compared to young nematodes. mtDNA lesions in old animals were 2.3-fold more than young animals (*p* = 0.04, Student’s *t*-test), *n* = minimum 9 independent experiments.

We first asked if mtDNA damage burden, as measured by our assay, increases with age in *C. elegans*. We thus compared mtDNA damage between young (day 4) and aged (day 14) *glp-1* nematodes. Under the conditions used, the *glp-1* strain had a mean lifespan of about 13 days in our laboratory, meaning that beyond day 14, worms starting to die rapidly (Figure 8A,B). We indeed observed a clear age-dependent increase in mtDNA lesions with age (Figure 5, *p* = 0.04, Student’s *t*-test). There was a 2.3-fold increase in mtDNA damage in old animals (1.09 DNA lesions per 10 kbp) compared to young animals (0.48 DNA lesions per 10 kbp). This is consistent with previous observations, by us and others, reporting age-dependent increases in DNA damage to both nDNA and mtDNA in aging animals (Halliwell and Aruoma, 1991; Ames et al., 1993; Mecocci et al., 1994; de la Asuncion et al., 1996; Chaubey et al., 2001; Hamilton et al., 2001; Dhawan et al., 2009; Gruber et al., 2011). That older animals carry higher mtDNA damage burden than younger animals is expected and is a perquisite for such damage to play a causative role in aging. However, it is also possible that mtDNA damage accumulation may be merely a consequence or even a symptom of aging. If damage burden is causatively linked to aging, we would expect that long-lived strains consistently show less mtDNA damage and that, conversely, mutations that increase damage to mtDNA should shorten lifespan. To test these hypotheses, we next determined mtDNA damage levels and lifespan in a few *C. elegans* mutant strains that are either known to be long-lived [insulin-like growth receptor (IGF) pathway mutant strain, *daf-2*] or in strains carrying mutations likely resulting in increased ROS-mediated damage (antioxidant system/ROS mutants).

### Does mtDNA Damage in Mutant Strains Correlate to Lifespan?

**Figure 6:**
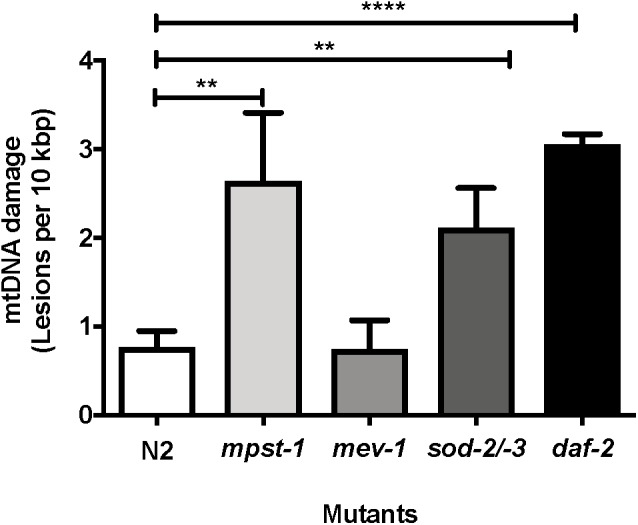
MtDNA damage in different mutant strains relative to young wild type N2 animals as measured using S-XL-qRT-PCR DNA damage assay. Relative to wild type animals, *mpst-1*, *sod-2/-3*, and *daf-2* mutant strains had 3.4-, 2.7-, and 4.0-fold higher mtDNA damage, respectively (*p* < 0.01 for both *mpst-1* and *sod-2/-3* mutants and *p* < 0.0001 for *daf-2* mutant, Student’s *t*-test). *mev-1* mutant *C. elegans* strain had no significant difference in mtDNA damage level compared to wild type N2 *C. elegans* (*p* > 0.05, Student’s *t*-test), *n* = minimum 3 independent experiments.

We first investigated *mpst-1* mutant animals. The *mpst-1* gene codes for a mitochondrial enzyme responsible for synthesizing hydrogen sulfide (H_2_S) (Miller and Roth, 2007; Qabazard et al., 2013). H_2_S has been shown to act as antioxidant and modulator of ROS production (Qabazard et al., 2013). We have previously shown that loss of *mpst-1* gene in *C. elegans* reduces H_2_S production, significantly elevates ROS production and reduces both lifespan and healthspan (Qabazard et al., 2013; Ng et al., 2018). Using our new assay and in agreement with our previous findings, we found that in short-lived *mpst-1* mutants, mtDNA damage was threefold higher than in wild type N2 animals and this increase was statistically significant (Figure 6, *p* < 0.003, Student’s *t*-test). In *mpst-1*, our data are therefore consistent with the assumption that high mtDNA damage contributes to shorter lifespan in mutants with defects in their control of ROS.

We then applied our assay to another mutant strain often considered to suffer from ROS related lifespan detriment. The, *mev-1* mutant strain was originally identified as a methyl-viologen (paraquat)-sensitive mutant strain (Ishii et al., 1990). However, later it was found that the gene *mev-1* encodes the enzyme succinate dehydrogenase cytochrome b, which is a key component of complex II in the mitochondrial electron transport chain (Ishii et al., 1998). The *mev-1* strain has decreased complex II activity (Ishii et al., 2011), is generally hypersensitive to oxidative stress, produces more ROS and suffers from increased oxidative damage and short lifespan (Adachi et al., 1998; Fong et al., 2017). However, despite having repeatedly been shown by us and others to produce more mitochondrial ROS and suffer from elevated oxidative damage, at least as assessed by protein carbonyl levels (Adachi et al., 1998; Fong et al., 2017), we did not detect any increase in mtDNA damage in short-lived *mev-1* mutants compared to N2 wild type nematodes (Figure 6, *p* = 0.96, Student’s *t*-test).

We next used our mtDNA damage assay to evaluate mtDNA damage levels in a mutant strain defective in a key mitochondrial ROS detoxification system. Superoxide dismutase (SOD) is an antioxidant enzyme that catalyzes dismutation of superoxide anions to hydrogen peroxide and oxygen (Van Raamsdonk and Hekimi, 2009; Gruber et al., 2011; Halliwell and Gutteridge, 2015). *C. elegans* has six isoforms of SOD, two of which (*sod-2* and *sod-3*) are mitochondrial MnSODs. GA480 is a *sod-2/sod-3* double knockout mutant strain lacking both of these mitochondrial forms of SOD. However, surprisingly, despite completely lacking mitochondrial SOD and being hyper-sensitive toward oxidative stress, this strain does not experience lifespan shortening (Doonan et al., 2008), probably due to compensatory suppression of mitochondrial metabolism (Gruber et al., 2011). We have previously shown that *sod-2/sod-3* double knockout mutant have significantly lower ROS production rate as evaluated by the DCF-DA assay (Gruber et al., 2011). Applying an earlier version of our mtDNA damage assay, we have previously found a trend toward elevated mtDNA damage in this strain but this trend did not reach significance (Gruber et al., 2011). Whether or not the lifespan of the GA480 strain is normal despite significantly elevated oxidative damage is therefore an important open question (Doonan et al., 2008; Honda et al., 2008; Gruber et al., 2011). We applied our new method to address this question and found a threefold increase in mtDNA damage relative to N2 wild type animals (Figure 6, *p* < 0.008, Student’s *t*-test). It therefore appears that animals of the SOD double mutant strain have normal lifespans, despite significantly higher damage to their mtDNA.

In summary, we found that only one mutant strain (*mpst-1*) shows both high mtDNA damage and short lifespan while one (*mev-1*) is short-lived despite normal mtDNA damage levels and lastly, one strain (*sod-2/sod-3* double mutants) has a normal lifespan despite significantly increased damage levels. These data therefore do not consistently support the notion that mtDNA damage level is mechanically linked to longevity.

We next explored a nematode mutant strain that is known to be long-lived (Kimura et al., 1997). Mutation in the IGF receptor, *daf-2* is one of the best studied and most efficacious single gene aging mutations in *C. elegans*. The *daf-2* mutants lacks a functional IGF receptor and lives much longer than the wild type N2 nematodes (Kenyon et al., 1993; Bansal et al., 2015). Studies have shown that the *daf-2*/IGF pathway regulates aging, and that mutation in *daf-2* decreases the formation of free-radicals and thus reduces protein oxidation (Yasuda et al., 1999; Mabon et al., 2009; Brys et al., 2010). Loss of *daf-2* also increases resistance to oxidative stress, most likely by activating expression of antioxidant and stress genes downstream of *daf-16*, including SODs (Brys et al., 2010). However, when we quantified the levels of mtDNA damage in *daf-2* mutants, we found that mtDNA damage levels were not lower than wild type N2 controls but were in fact threefold higher in *daf-2* mutants relative to wild type N2 controls and this elevation was statistically highly significant (Figure 6, *p* < 0.0001, Student’s *t*-test).

The above example shows that genetic perturbations that significantly and reproducibly extend lifespan are not necessarily associated with low or even normal mtDNA damage levels. While in some cases such as the *mpst-1* strain, mutation that cause increased ROS production may result in significantly increased damage levels and shortened lifespan, mtDNA damage levels were equally elevated in long-lived *daf-2* mutants as in *mpst-1* but without any apparent ill effect (Table 1). The lack of consistent impact of mtDNA damage level on aging rate (and *vice versa*) suggests that changes in mtDNA damage levels do not correlate with aging and lifespan in *C. elegans*.

**Table 1:** Comparison of the lifespan versus the damaged mtDNA level in *C. elegans* mutant strains relative to wild type N2 *C. elegans*.

Strain	Changes in survivorship relative to N2	Changes in mtDNA damage level relative to N2
*mpst-1*	Decreased	Increased
*mev-1*	Decreased	Normal
*sod-2/sod-3*	Normal	Increased
*daf-2*	Increased	Increased

*As expected, mpst-1 has short lifespan and high mtDNA damage level. Surprisingly, short-lived mev-1 mutant animals do not suffer from high level of damaged mtDNA. C. elegans lacking mitochondrial SOD, the sod-2/-3 double mutant animals did not experience reduced lifespan but had significantly higher mtDNA lesions. Unexpectedly, daf-2 long-lived mutants had high mtDNA lesions.*

### Does Elevated mtDNA Damage Shorten Lifespan?

In our mutant strain comparison, we found that there was no consistent relationship between differences in mtDNA damage and changes in lifespan. However, these effects were all measured in mutant strains, meaning that differences in mtDNA damage levels were the result of perturbations in endogenous processes that control signaling, ROS production/detoxification, DNA damage rate as well as DNA repair and turnover. In response to such perturbations, mutant strains may activate compensatory mechanisms potentially making them physiology significantly different from wild type animals. To more directly test the effect of mtDNA damage on lifespan, we therefore next asked the question whether exogenous damage to mtDNA shortens lifespan in adult *C. elegans*.

**Figure 7:**
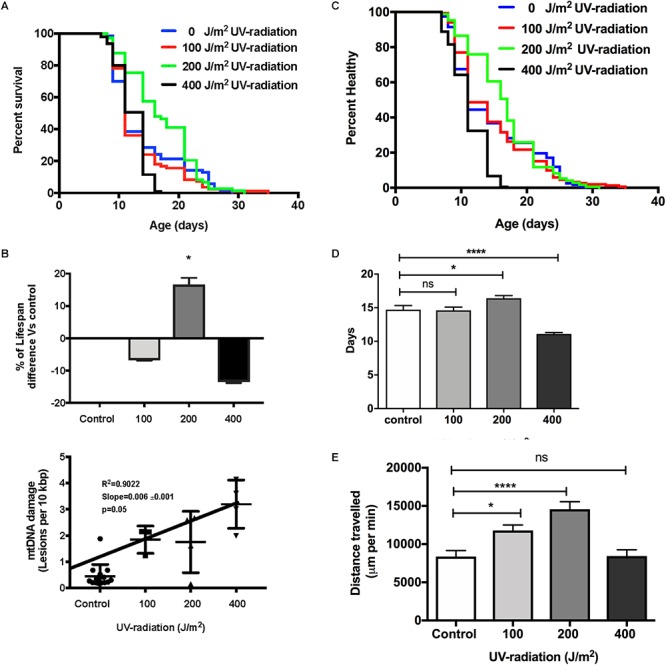
Dose-response study of UV-irradiated young *glp-1* nematodes. **(A)** Survival curves from each condition were compared to that of the non-irradiated control animals and analyzed using Log-rank (Mantel-Cox) test. The survival of nematodes irradiated with 100 J/m^2^ was not significantly different from the control. Lifespan of nematodes irradiated with 200 J/m^2^ was significantly extended, while exposure to 400 J/m^2^ significantly shortened the overall nematodes lifespan (*p* < 0.001). *n* = 200 worms per conditions**. (B)** Comparison of the percentage of lifespan difference and the mtDNA damage level of nematodes irradiated with 0, 100, 200, and 400 J/m^2^ UV-radiation. There was a trend toward dose dependent increase in damaged mtDNA in nematodes exposed to UV-radiation, interestingly, there was a hormetic lifespan extension effect in nematodes irradiated with 200 J/m^2^ UV-radiation compared to control animals (*p* < 0.05, survival curve comparison Log-rank (Mantel-Cox) test) **(C)** Overall, 200 J/m^2^ UV-irradiated animals were healthier (*p* < 0.0001, Log-rank (Mantel-Cox) test) **(D)** Relative to non-irradiated control animals, 200 J/m^2^ UV-radiation extended the mean healthspan by 12% but 400 J/m^2^ UV-radiation significantly shortened the mean healthspan of the nematodes by 25% (*p* < 0.05 and *p* < 0.001, respectively, Log-rank test with Bonferroni multiple comparisons test, OASIS 2), *n* = 200 worms per conditions **(E)** After UV-radiation, 100 and 200 J/m^2^ UV-irradiated animals traveled significantly more distance than control animals (*p* < 0.05 and *p* < 0.0001, respectively, One-way ANOVA with Bonferroni post-test). *n* = minimum 10 animals per condition.

To evaluate if increased mtDNA damage as induced by UV exposure and at the levels detected by our assay, has consequences on aging trajectories, we exposed *glp-1 C. elegans* to between 0 to 400 J/m^2^ of UV-radiation early in life. As mentioned earlier, compared to non-irradiated control animals, *C. elegans* exposed to both 100 and 200 J/m^2^ UV-radiation had fourfold higher mtDNA damage level and animals exposed to 400 J/m^2^ UV-radiation has sevenfold higher damaged mtDNA level. Comparing the survival of the untreated control animals to the nematodes exposed to 100, 200, and 400 J/m^2^ UV-radiation, there was no statistically significant decrease in lifespan in nematodes exposed to 100 J/m^2^ UV-radiation (Figure 7A,B, *p* > 0.05, Log-rank (Mantel-Cox) Test). In contrast, the lifespan of the nematodes irradiated with 400 J/m^2^ UV-radiation was significantly shorter than non-irradiated control animals (Figure 7A,B, *p* < 0.05, Log-rank (Mantel-Cox) Test). Surprisingly, in nematodes exposed to 200 J/m^2^ of UV-radiation, the mean survival was increased by 22% compared to non-irradiated control nematodes, suggesting a hormetic lifespan benefit at this level (Figure 7A,B, *p* < 0.05, Log-rank (Mantel-Cox) Test for survival curve and Log-rank test with Bonferroni multiple comparisons test, OASIS 2 for mean lifespan analysis). Whereas the healthspan of animals exposed to 100 J/m^2^ UV, measuring the motility as an indicator of health, was similar to the non-irradiated control animals [Figure 7C,D, *p* > 0.05, Log-rank (Mantel-Cox) test]. Exposure to 400 J/m^2^ UV-radiation, as expected, reduced overall health status [Figure 7C,D, *p* < 0.0001, Log-rank (Mantel-Cox) test]. Again, 200 J/m^2^ UV-radiation resulted in animals that were healthier on average and had extended mean healthspan compared to controls [Figure 7D, *p* < 0.05, Log-rank (Mantel-Cox) Test for survival curve and Log-rank test with Bonferroni multiple comparisons test, OASIS 2 for mean healthspan analysis]. Furthermore, animals irradiated with 200 J/m^2^ UV-irradiation had increased ability to sustain a high level of activity compared to controls or animals exposed to higher levels of UV (Figure 7E). In summary, animals irradiated with 200 J/m^2^ UV were healthier for a longer period of time, had a longer lifespan and traveled significantly more distances than animals from all other groups, including non-irradiated controls (Figure 7E, *p* < 0.0001, One-way ANOVA with Bonferroni’s post-test).

**Figure 8:**
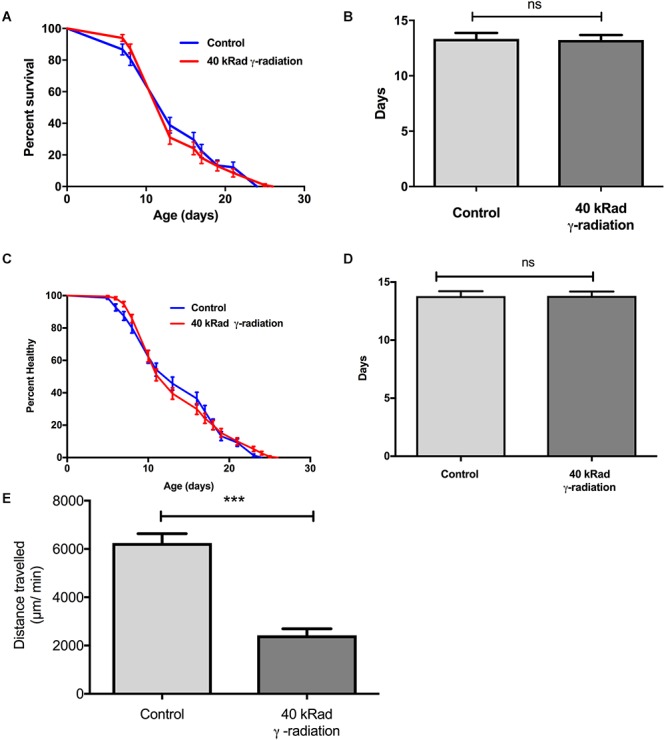
Dose-response study of young *glp-1* nematodes exposed to γ-radiation. The survival of day 4 young *glp-1* nematodes exposed to 40 kRad γ-radiation. **(A)** Survival curves from nematodes irradiated with 40 kRad γ-radiation are compared to that of the non-irradiated control animals. The lifespan of the 40 kRad γ-irradiated animals was not significantly shortened relative to the non-irradiated control animals [*p* > 0.05, Log-rank (Mantel-Cox) test]. **(B)** γ-radiation had no significant effect on the mean lifespan of the nematodes, both control and γ-irradiated animals have mean lifespan of 13 days (*p* > 0.05, Log-rank test with Bonferroni multiple comparisons test, OASIS 2). *n* = 200 worms per condition. Healthspan of the surviving day 4 *glp-1* nematodes exposed to 40 kRad **γ-**radiation**. (C)** As shown in the healthspan curve, the overall health status of the γ-irradiated animals was similar to control animals (*p* > 0.05, Log-rank (Mantel-Cox) Test for survival curve). **(D)** Both non-irradiated control nematodes and 40 kRad γ-irradiated nematodes had similar mean healthspan of 13.8 days (*p* > 0.05, Log-rank test with Bonferroni multiple comparisons test, OASIS 2). *n* = 200 worms per condition. **(E)** Average distance traveled by nematodes exposed to 40 kRad γ-radiation. γ-radiation significantly shortened the distance traveled by the nematodes compared to control animals. γ- irradiated nematodes traveled about 63% less than non-irradiated control animals (*p* < 0.0001, Student’s *t*-test). *n* = minimum 10 animals per condition.

We next evaluated if increased mtDNA damage as induced by γ-radiation had a more detrimental effect on aging trajectories than UV-induced damage. Given the preliminary results, we chose exposure to 40 kRad γ-radiation at day 4 of age. At this dosage γ-irradiation significantly elevated mtDNA damage levels to twofold higher (*p* < 0.01) in nematodes exposed to γ-radiation compared to untreated control animals (Figure 1B) and this amount of γ-radiation caused an observable increase in nDNA as measured by the Comet assay and also rendered WT animals sterile, confirming significant damage to both mtDNA and nDNA (Figure 4). To exclude the possibility that mtDNA damage levels due to γ-radiation were rapidly repaired, we have recently studied the mtDNA repair activity and found no significant repair of mtDNA damage within 24 h (Lakshmanan et al., 2018). Surprisingly, despite the fact that the animals had significantly and persistently higher mtDNA damage levels (Figure 1B), *C. elegans* exposed to 40 kRad of γ-radiation showed no significant lifespan shortening relative to untreated control animals (Figure 8A). The mean lifespan of the nematodes treated with 40 kRad γ-radiation was not significantly different relative to control animals (Figure 8B, *p* > 0.05, Log-rank test with Bonferroni multiple comparisons test, OASIS 2).

We then examined motility trajectory and speed of movement as indicators of overall health status of the muscular and nervous system. The health status of the γ-irradiated animals was similar to control animals, with similar mean healthspan at 13.8 days [Figure 8C,D, *p* > 0.05, Log-rank (Mantel-Cox) Test for survival curve and Log-rank test with Bonferroni multiple comparisons test, OASIS 2 for mean healthspan]. We then compared the average distance traveled within 15 min on day 4 of age between nematodes with or without pre-exposure to 40 kRad of γ-radiation. The typical distance traveled by γ-irradiated nematodes was markedly reduced relative to non-irradiated control animals (Figure 8E, *p* < 0.0001, Student’s *t*-test). Non-irradiated control nematodes traveled at 6400 μm per minute, while γ-irradiated nematodes traveled at about 2400 μm per minute (Figure 8E), suggesting some detrimental effects on performance at this level of radiation exposure, which, however, did not translate into shorter lifespan or accelerated aging. Despite significantly higher mtDNA damage in γ-irradiated animals, we observed no lifespan or healthspan shortening effects in γ-irradiated animals. This is surprising as we observed a clear fitness impairment (sterility and significantly reduced motility, Figure 2, 8C–E) in γ-irradiated animals.

## Discussion

Due to their short lifespan and the availability of powerful genetic tools, simple model organisms such as *C. elegans* are excellent tools to explore mechanism of aging and identify determinants of longevity. However, it is increasingly clear that the roles of free radicals, ROS and oxidative damage as determinants of lifespan and healthspan are complex (Lapointe and Hekimi, 2010; Ristow and Schmeisser, 2011; Stuart et al., 2014; Halliwell and Gutteridge, 2015). In the absence of carefully validated markers of oxidative damage, it can be challenging to fully understand the consequences of genetic, physical and pharmacological perturbations on damage and aging. Lifespan effects of perturbations designed to elevate oxidative damage can only be interpreted meaningfully if their effect on the relevant form of damage is determined at the same time. However, compared to biomarkers available for mammalian and clinical studies, tools to measure damage in nematodes are less well-validated. Despite its supposed importance, oxidative damage to mtDNA, for example, has not been widely reported in aging studies involving nematodes, mainly due to methodological challenges (Collins et al., 1996). Here, we have re-visited several *C. elegans* aging and stress challenge paradigms, applying our S-XL-qRT-PCR DNA damage assay to determine levels of damage to mtDNA, with the aim of evaluating the role of mtDNA damage in nematode aging.

Applying our assay to answer the question if mtDNA damage determines lifespan in nematodes, we first confirmed our previous observation that there is an increase in mtDNA damage with age in *C. elegans* (Gruber et al., 2011). This is a key expectation of the mFRTA which suggests that old animals should carry higher damage burden, especially in mitochondria, than young ones (Fraga et al., 1990; Stadtman, 1992; Yasuda et al., 2006; Gruber et al., 2011). Several studies in humans (Mecocci et al., 1993), rats and mice (Hamilton et al., 2001), and flies (Agarwal and Sohal, 1994) have, similarly, found detectable mtDNA damage accumulation with age, although using different methods. However, these observations alone do not support a causative role of mtDNA in aging. If mtDNA was indeed limiting to lifespan, damage to mtDNA would be expected to be low in long-lived animals and perturbations that elevate mtDNA damage burden should result in shorter lifespan. To test if this was the case, we next determined mtDNA damage levels and lifespan in *C. elegans* mutant strains that are either known to be long-lived (*daf-2*) or that carry mutations thought to increase ROS-mediated damage (mutations affecting antioxidant system or ROS production). Comparing damage of these strains to wild type, we found that only one strain, *mpst-1*, exhibited both high mtDNA damage burden and short lifespan. This strain carries a *mpst-1* null deletion allele, resulting in reduced endogenous H_2_S production and this causes both increased oxidative damage and short lifespan (Qabazard et al., 2013). There are known links between mitochondrial function and mtDNA damage (Cui et al., 2012; Modis et al., 2013), but there are also suggestions that H_2_S production by mitochondrial 3-MST may control mitochondrial function directly (Modis et al., 2013). Thus, it is unclear whether the observed lifespan shortening directly related to elevation of damaged or more generally to dysregulation in mitochondria.

Testing another mutant strain with abnormal mitochondrial ROS production, *mev-1*, we found no elevation of mtDNA damage level, despite the fact that the animals are short-lived and suffer from elevated oxidative stress to protein (Adachi et al., 1998; Ishii et al., 1998; Fong et al., 2017). *Mev-1* mutants are known to carry a defect in an ETC subunit of Complex II and suffer from mitochondrial dysfunction and elevated oxidative damage to protein as evaluated by protein carbonyl content (Adachi et al., 1998). We have also recently shown that there are further metabolic deficits in *mev-1* mutants which may contribute to their shorter lifespan (Senoo-Matsuda et al., 2001, 2003; Fong et al., 2017). These data are consistent with the notion that the short lifespan of *mev-1* mutants is due to increased damage to mitochondria, resulting in impaired mitochondrial function. However, our data suggest that this does not involve or require increased damage to mtDNA *per-se*. Finally, we applied our assay to determine mtDNA damage burden in the *sod-2/sod-3* double mutant strain, finding measurably elevated damage despite normal lifespan. This result, again, is inconsistent with the expectations of the mFRTA where significant elevation of mtDNA damage is expected to negatively impact lifespan of the mutant nematodes (Yang et al., 2007; Doonan et al., 2008; Honda et al., 2008; Van Raamsdonk and Hekimi, 2010; Gruber et al., 2011). Previously, Van Raamsdonk and Hekimi (2009) has reported that deletion of MnSOD in *C. elegans* can even increase the lifespan of the nematodes. Others have reported that mice heterozygous for MnSOD deletion also suffer from elevated mtDNA damage but these animals also do not have shortened lifespan (Van Remmen et al., 2003). The lifespan of mice homozygous for loss of MnSOD by contrast are severely shortened although there are no data on mtDNA damage levels in these animals (Huang et al., 2001; Kokoszka et al., 2001; Hinerfeld et al., 2003). These mice lacking mitochondrial SOD also suffered from accumulation of high lipid peroxidation products and decreased mitochondrial respiration (Li et al., 1995; Kokoszka et al., 2001). Finally, mice homozygous for loss of Cu/ZnSOD do experience shorter lifespan and increased mtDNA damage levels (Elchuri et al., 2005). These data therefore support the notion that DNA damage to nDNA and/or mtDNA, within a relatively wide physiologically range, is not a direct determinant of individual longevity while severe damage above some threshold will, of course, become detrimental. A previous study that is often cited in support of the mFRTA indeed found the expected inverse relationship between lifespan and mtDNA (but not nDNA) burden, when comparing DNA damage levels between animal species with maximum lifespans ranging from 2 to 46 years (Barja and Herrero, 2000). Also, wide comparative studies conducted by Lehmann et al. (2008, 2013) and Toren et al. (2016) showed that mtDNA GC content is a strong and independent determinant of mammalian longevity. Remarkably, the mtDNA GC content did not correlate with another determinant of mammalian longevity, resting metabolic rate (Lehmann et al., 2008, 2013; Toren et al., 2016). However, in contrast to this study, here we have investigated the impact of changes in mtDNA damage on lifespan within a single species. Our data suggest that over a relatively wide range of values, changes in mtDNA damage burden do not translate into differences in lifespan, at least in nematodes. Similar logical criteria, as in our study, were used for the analysis of nDNA damage in aging (Moskalev et al., 2013; Yanai and Fraifeld, 2018). Interestingly, using such criteria for the analysis of nDNA damage in aging, Moskalev et al. (2013) concluded that the existing data is insufficient to prove that nDNA damage plays a casual role in aging.

We next evaluated damage burden in long lived *daf-2* mutants (Kimura et al., 1997). Consistent with the expectation of the mFRTA, *daf-2* have been reported to be more resistant to oxidative stress (Honda and Honda, 1999), produce fewer free radicals and to carry lower protein carbonyl contents (Brys et al., 2010). Another study by Yasuda et al. (1999), also reported reduced accumulation of protein carbonyls in long-lived *daf* mutants of *C. elegans*. However, surprisingly, we found that, despite their long lifespan, *daf-2* mutants experience a significant elevation in mtDNA damage level compared to shorter-lived WT animals. Interestingly, *daf-2* mutants also have lower metabolic activity than wild type (Van Voorhies and Ward, 1999) and have been reported to have decreased protein turnover (Yasuda et al., 1999; Depuydt et al., 2016) and slower protein aggregation (David et al., 2010; Buisset-Goussen et al., 2014). Together, these data suggest that improved proteostasis and decreased translation may be associated with lifespan benefits in *C. elegans* (Syntichaki et al., 2007; Essers et al., 2015; Walther Dirk et al., 2015; Uno and Nishida, 2016).

The lack of consistent impact of mtDNA damage levels on lifespan (and *vice versa*) suggests that changes in mtDNA damage levels do not trivially correlate with lifespan. However, one limitation with the data discussed above is that they are all determined in animals subject to genetic perturbation and such perturbations may result in potentially confounding compensatory mechanism (El-Brolosy and Stainier, 2017).

In order to more directly test the relevance of mtDNA damage in the context of lifespan determination, we therefore introduce damage to mtDNA directly by exposing young *C. elegans* to UV- or γ-radiation. Sufficiently high levels of UV-radiation cause extensive mtDNA damage and this indeed shortened *C. elegans* lifespan. However, we found that lower levels of this stressor still significantly increase mtDNA damage but without causing significant detriments and that some levels even resulted in lifespan extension and healthspan improvements. This is consistent with the concept of hormesis; that exposure to mild stress, through evoking adaptive responses and strengthening stress defense mechanisms can lead to lifespan extension (Gruber et al., 2015). However, it is worth noting that in our experiments, even under conditions where UV damage results in hormetic benefits, damage remained detectably elevated, even on the day following exposure (Lakshmanan et al., 2018). The lack of evidence for a tight relationship between mtDNA damage burden and lifespan in *C. elegans* is consistent with our recent finding that, most likely due to the short lifespan of nematodes, mtDNA deletion do not accumulate with age in *C. elegans* (Lakshmanan et al., 2018).

Using γ-radiation instead of UV light, we found that exposure to 40 kRad of γ-radiation also significantly elevated damaged to mtDNA but this, again, did not cause any lifespan detriments. Fully developed, adult *C. elega*ns consist of non-dividing somatic cells (Flemming et al., 2000; Bailly and Gartner, 2011) and are therefore able to survive high levels of radiation. For comparison, 40 kRad of γ-radiation is approximately 100 times higher than the 0.4 kRad radiation dose that is expected to cause 50% mortality in humans (Mole, 1984). This means that animals exposed to 40 kRad of γ-radiation should have sustained significant damage, a fact also confirmed by the observation that this level of radiation causes a significant increase in mtDNA damage, nDNA strand breaks and renders animals sterile (Buisset-Goussen et al., 2014) However, despite of this, the γ-radiation challenge was ineffective in shortening nematode lifespan. Interestingly, conditions associated with elevated damage to mtDNA, including γ-radiation, were consistently associated with reduced mitochondrial metabolism (Allen and Sohal, 1982; Chung et al., 2001). Conditions where mtDNA damage is high therefore appear to be associated with impaired energy metabolism but not necessarily with shortened lifespan in *C. elegans*. From these results it appears that, within a relatively wide range, oxidative damage to mtDNA is not a limiting factor for longevity, at least in nematodes.

## Author Contributions

JG and LFN designed the experiments. LFN and LTN performed experiments and analyzed results. LFN, LTN, and JG wrote the manuscript. JG conceived and supervised the project. LFN, LTN, MvB, BH, and JG contributed critical comments and corrections and have approved the manuscript.

## Conflict of Interest Statement

The authors declare that the research was conducted in the absence of any commercial or financial relationships that could be construed as a potential conflict of interest.
